# Assessing the Influence of Propylthiouracil and Phenytoin on the Metabolomes of the Thyroid, Liver, and Plasma in Rats

**DOI:** 10.3390/metabo13070847

**Published:** 2023-07-14

**Authors:** Zhipeng Wang, Sven-Bastiaan Haange, Volker Haake, Maike Huisinga, Hennicke Kamp, Roland Buesen, Kristin Schubert, Sebastian Canzler, Jörg Hackermüller, Ulrike Rolle-Kampczyk, Martin von Bergen

**Affiliations:** 1 Department of Molecular Systems Biology, Helmholtz-Centre for Environmental Research—UFZ, 04318 Leipzig, Germany; 2 BASF Metabolome Solutions GmbH, 10589 Berlin, Germany; 3 Experimental Toxicology and Ecology, BASF SE, 67056 Ludwigshafen, Germany; 4 Department of Computational Biology, Helmholtz-Centre for Environmental Research—UFZ, 04318 Leipzig, Germany; 5 Department of Computer Science, University of Leipzig, 04109 Leipzig, Germany; 6 Institute of Biochemistry, Faculty of Biosciences, Pharmacy and Psychology, University of Leipzig, 04103 Leipzig, Germany; 7 German Centre for Integrative Biodiversity Research, (iDiv) Halle-Jena-Leipzig, 04103 Leipzig, Germany

**Keywords:** thyroid hormones, propylthiouracil, phenytoin, metabolomics, hypothyroidism

## Abstract

The thyroid hormones (THs) regulate various physiological mechanisms in mammals, such as cellular metabolism, cell structure, and membrane transport. The therapeutic drugs propylthiouracil (PTU) and phenytoin are known to induce hypothyroidism and decrease blood thyroid hormone levels. To analyze the impact of these two drugs on systemic metabolism, we focused on metabolic changes after treatment. Therefore, in a rat model, the metabolome of thyroid and liver tissue as well as from the blood plasma, after 2-week and 4-week administration of the drugs and after a following 2-week recovery phase, was investigated using targeted LC-MS/MS and GC-MS. Both drugs were tested at a low dose and a high dose. We observed decreases in THs plasma levels, and higher doses of the drugs were associated with a high decrease in TH levels. PTU administration had a more pronounced effect on TH levels than phenytoin. Both drugs had little or no influence on the metabolomes at low doses. Only PTU exhibited apparent metabolome alterations at high doses, especially concerning lipids. In plasma, acylcarnitines and triglycerides were detected at decreased levels than in the controls after 2- and 4-week exposure to the drug, while sphingomyelins and phosphatidylcholines were observed at increased levels. Interestingly, in the thyroid tissue, triglycerides were observed at increased concentrations in the 2-week exposure group to PTU, which was not observed in the 4-week exposure group and in the 4-week exposure group followed by the 2-week recovery group, suggesting an adaptation by the thyroid tissue. In the liver, no metabolites were found to have significantly changed. After the recovery phase, the thyroid, liver, and plasma metabolomic profiles showed little or no differences from the controls. In conclusion, although there were significant changes observed in several plasma metabolites in PTU/Phenytoin exposure groups, this study found that only PTU exposure led to adaptation-dependent changes in thyroid metabolites but did not affect hepatic metabolites.

## 1. Introduction

Thyroid hormones (THs) triiodothyronine (T3) and thyroxine (T4) regulate many important biological processes in the body, including the normal development of tissue, cellular metabolism, cell structure, and membrane transport [1]. THs are synthesized and secreted from thyroid follicular cells in the thyroid gland either as the active T3 or in the inactive T4 form [2]. After synthesis in the thyroid, T3 and T4 enter the bloodstream, reaching the different target organs, including the brain and liver. The conversion of T4 to the active T3 form is also facilitated in other tissue such as the kidney, brown adipose tissue, pituitary gland, liver, brain, and heart by 5′-deiodinase type 1 (D1) or 5′-deiodinase type 2 (D2) [3], which are the primary sources of circulating T3. In the liver, T3/T4 can be metabolized species-specifically to either glucuronide or sulfate conjugates and then excreted through the bile duct into the intestine [4]. T3 can also be deactivated by 5-deiodinase type 3 (D3) in the skin, vascular tissue, and placenta [5].

The synthesis of THs is regulated by the thyroid-hypothalamus axis [5]. Circulating T4 reaches the hypothalamus and is converted by D2 to the active T3, inhibiting the secretion of thyrotropin-releasing hormone (TRH). Conversely, TRH secretion can be activated by the signal transducer and activator of transcription STAT3, which is activated by phosphorylation stimulated by leptin, produced in peripheral fat tissue [5]. The released TRH reaches the anterior pituitary gland stimulating the release of thyroid-stimulating hormone (TSH). TSH circulates to the thyroid, where it binds to the G-protein coupled with the TSH receptor, which mediates the expression of genes and functions involved in THs synthesis [6], thereby increasing THs production and release.

Disturbances in TH homeostasis are linked to thyroid nodules, thyroiditis, hyperthyroidism, hypothyroidism, autoimmune thyroid disease, and thyroid cancer [7]. Hypothyroidism, the overt deficiency of THs, can cause several symptoms, such as fatigue, lethargy, cold intolerance, weight gain, and even goiter and death if untreated [2]. Hyperthyroidism is characterized by excessive production of thyroid hormones (THs) and is typically associated with symptoms such as weight loss, heat intolerance, anxiety, and muscle tremors [8,9].

Several drugs are known to influence the TH levels within the body. Propylthiouracil (PTU) directly affects the thyroid by inhibiting the thyroid peroxidase, and deiodinase type 1 is a commonly used antithyroid drug to treat hyperthyroidism [3,10]. The overt dosage of PTU administration leads to primary hypothyroidism. Phenytoin, a potent anticonvulsant used to treat and prevent generalized seizures, is a further drug influencing TH levels [11]. It has been associated with hypothyroidism [12], though it seems to function indirectly by not acting on the thyroid but by increasing the THs clearance in the liver [13].

Researchers have made considerable efforts to elucidate the toxic effects of drug-induced hypothyroidism from various perspectives and methods. For example, Edgar et al. found that compared to thyroidectomy-induced hypothyroidism, methimazole-induced hypothyroidism, an antithyroid drug, can cause cellular damage in several organs, such as the liver, lung, and kidney [14]. Meanwhile, Wu et al. used an untargeted metabolomics approach to identify 13 potential biomarkers related to sphingolipid metabolism, fatty acid transportation, phospholipid metabolism, and phenylalanine metabolism in rat serum after propylthiouracil administration [15]. They also systematically investigated the urinary metabolic profiling in three classic hypothyroidism models, i.e., methimazole-, propylthiouracil-, and thyroidectomy-induced hypothyroidism, and identified several metabolites related to energy metabolism, amino acid metabolism, sphingolipid metabolism, and purine metabolism [16]. In another study, Hennicke et al. conducted a metabolomics study on phenytoin-administered rat plasma samples. They found that phenytoin-induced toxicity can cause liver enzyme induction and liver toxicity, even though liver histopathology revealed slight centrilobular hepatocellular hypertrophy [17]. However, no systematic metabolomics studies have been carried out at the organ level, particularly in the thyroid gland and the liver. Changes in the molecular metabolome level induced by alterations in TH levels facilitated by PTU and phenytoin treatment are still not fully understood. Therefore, we investigated the influence of the two drugs PTU and phenytoin on the metabolome of the different organs involved in regulating TH levels. Specifically, we investigated the metabolomes from the thyroid tissue, where T3 and T4 are synthesized; the liver tissue, where THs are metabolized before excretion; and plasma, where systemic effects on the metabolome can be investigated. In addition, we investigated these metabolic changes induced by PTU and phenytoin administration in a dose- and time-dependent manner. The selection of the drug dosage administered was based on previous studies [17,18,19,20,21], with low doses known to induce toxic effects while high doses are known to cause known severe toxicity. Finally, we aimed at determining if any metabolic changes were reversible after ceasing administration of the drugs. The study design facilitated identifying metabolic changes in a concentration and time-dependent manner and which of the detected metabolic changes were reversible.

## 2. Materials and Methods

### 2.1. Animals and Maintenance Conditions

All animals were kept by BASF (Germany) according to their standard protocols. Ten animals for each treatment group were split into two separate cages. Humidity levels between 45–65% and 15 air changes per hour were set up, as well as 12 h of light from 06:00–18:00 h. The food used was ground Klibamaintenance diet mouse/rat “GLP”, meal, supplied by Granovit AG (Kaiseraugst, Switzerland). Food and drinking water (from water bottles) were available ad libitum. The animals were checked daily for abnormal clinical signs, and the clinical observations were documented for each affected animal. Animals were identified with ear tattoos. This study was performed in an *AAALAC*-approved laboratory following the German Animal Welfare Act (version of 2013) and the effective European Council Directive 2010/63/EU (2010). This study was approved by the local authorizing agency for animal experiments (Landesuntersuchungsamt Rheinland-Pfalz, Koblenz, Germany) as referenced by the approval number 23177-07/G 19-3-061.

### 2.2. Substances Administration and Samples Collection

The rat strain RjHan:WI was used. Animals were supplied from Janvier Labs, CS 4105 Le Genest-Saint-Isle, F-53941 Saint-Berthevin Cedex. The study was conducted using three subsets, A, B, and C, which represented three planned exposure groups: short-term (2 weeks), long-term (4 weeks), and long-term (4 weeks) with a recovery period (2 weeks). The age at the start of the administration period was 16 weeks (subset A), 14 weeks (subset B), and 12 weeks (subset C). The animals were allocated to the test groups before the start of the administration period based on their weight. The weight variation of the animals did not exceed 20 percent of the mean weight. At the beginning of the administration period, the male animals had a mean weight range of 477.0 to 502.6 g. Each test group consisted of 10 male animals (with 5 animals per cage). Test substances PTU (6-Propyl-2-thiouracil, Sigma-Aldrich, Taufkirchen, Germany) and phenytoin (Sigma-Aldrich, Taufkirchen, Germany) were weighed and mixed with a small amount of food in a laboratory mixer and reached the desired concentration by further mixing with the food.

In the present study, 300 ppm and 2400 ppm were chosen as the low and high doses for phenytoin, whereas 5 ppm and 50 ppm were selected as the low and high doses for PTU. The substance intake was monitored weekly to calculate the mean daily intake and recorded as test substance intake (mg/kg/day). Food intake data were collected for each group since the animals were housed together. To obtain this measure, the following formula was used: Daily food intake (g/day) × nominal substance concentration in diet (mg test item/g vehicle) ÷ Average body weight during the food consumption interval (kg). The calculated daily intake of PTU in subsets A (2 weeks), B (4 weeks), and C (6 weeks) were 0.2 and 2.2 mg/kg/day for low and high-dose groups, respectively. For phenytoin, the intake was 15 mg/kg/day in the low-dose groups in subsets A, B, and C. In the high-dose groups of subsets A, B, and C, the intake was 96, 111, and 121 mg/kg/day, respectively.

Body weight was determined before the administration period to randomize the animals into groups. During the administration period, the body weight was determined on day 0 (the start of the administration period) and after that at weekly intervals.

In the morning (08:00–10:00 h), blood samples were taken from fasted animals by puncturing the retrobulbar venous plexus under isoflurane anesthesia (3.5–4% Isoflurane in air with a flow rate of 160 L/h). Anesthesia was confirmed by a lack of eyelid reflex, lack of intertoe reflex, and flat respiration. Blood sampling and examination were carried out in a randomized sequence (with randomization being compiled with a computer). From each animal, 1 mL of blood was collected with either EDTA-K3 (potassium salt) as an anticoagulant (10 μL of a 10% solution) for plasma metabolome profiling or EDTA-free for serum TSH/T4/T3 concentration measurement. The samples were centrifuged at 14,000× *g* under 4 °C, and the plasma was separated. The blood was sampled and prepared in original 2 mL Eppendorf tubes. The preparation of the samples was conducted under cooling. All samples were stored covered with an N_2_ atmosphere at −80 °C. Blood samples were taken on 2 consecutive days sampling 5 animals per group and day for subset A on study days 15/16, subset B on study days 28/29, and subset C on study days 42/43.

The serum concentrations of TSH were determined by direct, competitive radioimmunoassay (RIA), using rat-specific TSH RIA kits (Institute of Isotopes Co., Ltd., Budapest, Hungary, cat.no. RK-554) and a Gamma-Counter (LB 2111, Berthold, Germany) and evaluated with the LBIS501 (version: 4.00) software of the instrument producer. The TSH control kit used is called Control Set rTSH (Trilevel; cat-no RK-554Cset, Izotop, Budapest, Hungary). An automatic smoothed cubic Spline curve fit method was applied (smoothing factor 0.125). Acceptance criteria for the standard curve were a total count of >10,000 cpm and a non-specific binding of <5%. The lowest quantifiable value (LOQ) is 1 μg/L. Values above the linear range (20.80. intercept of the curve) were repeated with diluted samples. At least two levels of quality control were applied in each test run. Intra- test-run and between-test-run precision of TSH values in the range of 3.7–33.5 μg/L were below 16.3 (% coefficient of variation, values from kit provider).

T4 ELISA kits (DRG Instruments, Marburg, Germany, cat.no. EIA-4568) were measured with a Sunrise MTP-reader (Tecan AG, Maennedorf, Switzerland) and evaluated with the Magellan-Software (version: 7.3) of the instrument producer. Bilevel controls were provided within the test kits. A Four-Parameter-Marquardt curve fit was applied. Acceptance criteria for the standard curve were a maximum OD of at least 1.5 and a curve fit correlation of r = 0.999. The linear range of the standard curve is defined as a 10–90 intercept of the curve. Values above the range were repeated with diluted samples. The lowest quantifiable value (LOQ) is 15 nmol/L (7.5 nmol/L for fetuses and PND4 pups). Two levels of quality control were applied on each microtiter plate. Intra-test run and between-test run precision of T4 values in the range of 23–179 nmol/L were below 12.0 (% coefficient of variation, values from in-house validation).

T3 ELISA kits (Diagnostic Biochem Canada Inc., London, Ontario, Canada, cat.no. CAN-T3-4220) were measured with a Sunrise MTP-reader (Tecan AG, Maennedorf, Switzerland) and evaluated with the Magellan-Software (version: 7.3) of the instrument producer. Bilevel controls were provided within the test kits. A Four-Parameter-Marquardt curve fit was applied. Acceptance criteria for the standard curve were a maximum OD of at least 1.2 and a curve fit correlation of r = 0.999. The linear range of the standard curve is defined as a 10–90 intercept of the curve. Values above the range were repeated with diluted samples. The lowest quantifiable value (LOQ) is 0.3 nmol/L. Two levels of quality control were applied on each microtiter plate. Intra-test run and between-test run precision of T3 values in the range of 1.4-2.2 nmol/L were below 11.3 (% coefficient of variation, values from in-house validation).

All animals were sacrificed by decapitation under isoflurane anesthesia (as described above). The exsanguinated animals were necropsied, i.e., a post-mortem examination took place by opening the body and assessing visually (gross necropsy). Organs were removed and weighted before fixation. The liver and left thyroid gland were preserved in a 4% neutral buffered formaldehyde solution and embedded in paraffin. Sections of 4 μm were stained with hematoxylin and eosin (H&E) and examined by light microscopy [22]. From the right thyroid gland (with parathyroid gland), serial sections were prepared with the cryostat and split alternating in 3 separate samples for the different omics experiments.

### 2.3. Targeted Metabolomics for Plasma Samples

The standardized protocol for mass spectrometry-based targeted metabolite profiling of blood plasma samples is described below [23,24]. All 10 harvested replicates from each treatment group were input into plasma metabolomics experiments.

Targeted metabolites were measured from 60 μL rat plasma by extracting with a mixture of methanol, dichloromethane, water, and toluene (93:47:16,5:1, *v*/*v*) buffered with ammonium acetate using a ball mill ( OMNI INTERNATIONAL, 935 Cobb Place Blvd, Kennesaw, GA, USA). Internal standards were added to the extraction mixture to enhance reproducible analysis. After centrifugation (12,000 rpm 10 min@ 12 °C), an aliquot of the extract was subjected to LC-MS/MS analysis using reversed-phase and hydrophilic interaction liquid chromatography (ZIC—HILIC, 2.1 × 100 mm, 3.5 μm, Supelco, Merck KGaA, Darmstadt, Germany) followed by MS/MS detection (AB Sciex QTrap 6500+) using the positive and negative ionization mode. For reverse-phase high-performance liquid chromatography (RP-HPLC, Ascentis Express C18, 5 cm × 2.1 mm, 2.7 μm Supelco), gradient elution was performed with water/methanol/0.1 M ammonium formate (1:1:0.02 *w*/*w*) and methyl-tert-butylether/2-propanol/methanol/0.1 M ammonium formate (2:1:0.5:0.035 *w*/*w*) with 0.5% (w%) formic acid (0 min 100% A, 0.5 min 75% A, 5.9 min 10% A). HILIC gradient elution was performed with acetonitrile with 1 vol-% water and 0.2 vol-% acetic acid (A) and 0.007 M ammonium acetate with 0.2 vol-% acetic acid (B) (0 min 100% A, 5 min 10% A, 600 μL/min). 

A second aliquot of the extract was mixed with water (3.75:1, *v*/*v*), resulting in phase separation. Both phases were analyzed with gas chromatography-mass spectrometry (GC7890-5975 MSD, Agilent Technologies, Santa Clara, CA, USA) after derivatization, as described in Grossmann et al. [25]. Briefly, the non-polar fraction was evaporated to dryness and treated with a mixture of 130 μL of dichloromethane, 40 μL of hydrochloric acid, 310 μL of methanol, and 20 μL toluol followed by heating (2 h, 100 °C) and evaporation to dryness. The polar and non-polar fractions were further derivatized with O-methyl-hydroxylamine hydrochloride (20 mg/mL in pyridine, 50 μL for 1.5 h at 60 °C) to convert oxo-groups to O-methylomes. Subsequently, 50 μL of the silylating agent *N*-methyl-*N*-(trimethylsilyl)-2,2,2-trifluoroacetamide (MSTFA), and 10 μL internal standard mix was added, and the derivatization was carried out for 30 min at 60 °C. The polar fraction was chromatographed on a DB-XLB column (30 m × 0.25 mm × 0.25 μm) using an oven temperature program (70 °C for 2 min, 50 °C/min up to 100 °C, 8 °C/min up to 200 °C, 14 °C/min up to 340 °C for 5 min, 100 °C/min down to 70 °C). The non-polar fraction was chromatographed on an HP-5 MS column (30 m × 0.25 mm × 0.25 μm) using an oven temperature program (70 °C for 3 min, 50 °C/min up to 130 °C, 10 °C/min up to 340 °C for 9 min, 120 °C/min down to 70 °C).

All the samples were analyzed once in a randomized analytical sequence design to avoid artificial results concerning analytical shifts. For GC-MS and LC-MS/MS profiling, data were normalized to the median of reference samples (“rat-ultra pool”). The rat-ultra pool reference material is used as standard in BASF rat plasma studies. It was generated by pooling blood plasma from a large number of animals. This material was aliquoted and freeze-dried, and aliquots are measured in parallel to the study samples to serve as a standard reference for inter- and intra-instrumental variation. A total of 495 metabolites was analyzed using the single peak signal of the respective metabolite and a normalization strategy according to the patent WO2007012643A1 [26], resulting in ratio values representing the metabolite change in treated versus control animals. Of those 495 analyzed metabolites, 454 are chemically identified, and 41 are structurally unknown. It should be noted that only 420 unique metabolites were considered for further data analysis.

Steroid hormones, catecholamines, and their metabolites were measured from separate plasma aliquots, 50 μL each, by online SPE-LC–MS/MS (Solid phase extraction-LC–MS/MS, Symbiosis Pharma Pro Online SPE UHPLC System, Spark Holland) in positive electrospray mode using stable-isotope-labeled internal standards [27,28].

Before steroid analysis, the samples were extracted with 500 μL *MTBE* (methyl t-butyl ether). After evaporation, the residue was derivatized by dissolving it in 80 μL of acetonitrile/water (9:1) with ultrasonication for 3 min, adding 40 μL of dansyl chloride (1 mg/mL in acetonitrile) and 40 μL of NaHCO_3_ (0.2 mol/L), then shaking for 20 min at 60 °C at 400 rpm (Thermomixer) and adding 75 μL of water after cooling it to ambient temperature. Chromatography was performed with a YMC Triart C18 ExRs plus 50 × 2.1 mm × 3 μm HPLC Column at 30 °C using 5% acetonitrile in water with 0.1% formic acid (*w*/*w*/*w*) (A) and acetonitrile with 0.1% formic acid (*w*/*w*) (B) (0 min 5% B, 5.5 min 65% B, 8 min 100% B, 9.5 min 100% B, 600 μL/min).

For catecholamine analysis, 50 μL of 0.01 M HCl (internal standard solution) was added, and proteins were removed by membrane filter tube centrifugation for 45 min at 13,000 rpm, 4 °C. An amount of 50 μL of each of NaHCO_3_ (0.2 mol/L), dansyl chloride (2 mg/mL in acetone), and methanol, was added to the filtrate and kept for 12 h at 4 °C before analysis. Chromatography was performed with an ODS 7 pH, 60 mm × 2 mm, 4 μm HPLC Column (Alltech Grom) at 30 °C using 5% acetonitrile in water with 0.5% formic acid (*w*/*w*/*w*) (A) and acetonitrile with 0.5% formic acid (*w*/*w*) (B) (0 min 40% B, 4 min 52% B, 4.17 min 70% B, 7 min 75% B, 11.67 min 90% B, 12 min 90% B, 300 μL/min).

### 2.4. Tissue Metabolites Extraction

In line with other omics layers studies, only 5 out of 10 animals from each treatment group were selected for tissue metabolomics experiments.

Due to the low stability of most tissue metabolites, the received tissue materials were immediately kept at −80 °C until the start of the experiment. The samples were transferred into a precooled freezer box that was already cooled to −80 °C when extracting metabolites. For liver samples, approximately 100 mg wet-weight materials were prepared, and weight was recorded by using an analytical balance at room temperature (RT) and transferred into a new Eppendorf tube. Due to the limited thyroid gland size, all received thyroid gland materials were weighed, recorded, and transferred into a new Eppendorf tube. The prepared tissue materials were mixed with a 5X a metabolite extracting solution of 50% acetonitrile and 50% water. With 3–5 steal balls and a tissue slicer for 10 min at 30 Hz, the separated tissue metabolites were then dissolved in an extraction solution. After 2 min of centrifugation at 14,000 rpm, the metabolites containing supernatant were recovered into a new Eppendorf tube and stored at −80 °C for future metabolite quantification experiments. It should also be noted that we used 5 out of 10 replicates for tissue metabolomics.

### 2.5. Targeted Metabolomics for Thyroid/liver Samples

The targeted metabolites quantification was conducted by using the MxP Quant 500 kit (Biocrates Life Science, Innsbruck, Austria), which includes the absolute quantification of 630 metabolites, covering 26 biochemical classes.

Further sample processing steps and measurement tasks were executed following the manufacturer’s instructions. Briefly, the sample preparation was done on the provided 96-well plate. A total of 7 calibration standard levels and 3 different quality control standards (QC1, QC2, QC3) were part of the Kit. Additional 4 QC2 samples were distributed over the plate. All other remaining wells were left for the current project samples. The samples were added to an adsorption pad system in the 96-well plate and dried for 30 min under nitrogen flow. Followed by derivatization using 50 μL 5% PITC (phenylisothiocyanate) for 60 min before 60 min drying under nitrogen flow, 300 μL extraction solvent (5 mM ammonium acetate in methanol) was added and shaken for 30 min. The samples were then eluted into the lower well by centrifugation for 30 min at 450 rpm. The extracted elutes were separated into two parts and diluted for FIA and LC measurement.

The metabolite measurements were processed in the QTRAP 5500 system (AB SCIEX) equipped with the LC 1290 Infinity system (Agilent). As for the FIA part, neither oven temperature nor columns were used, and only solvent B (290 mL methanol + 1 ampule FIA Mobile Phase Additive) was used for the injection. The flow rate was stabilized at 0.03 mL/min for 1.6 min, increased to 0.2 mL/min for 0.8 min, maintained for 0.2 min, and then decreased down to 0.03 mL/min in 0.2 min. The compound-specific MRM transitions were determined in positive ionization mode. For the LC part, an MxP Quan 500 Column System (Biocrates Life Science) was equipped for use and heated by setting the oven temperature to 50 °C.

The substance analysis via specific MRM transitions and LC was split into one part measured in positive (LC1) and another in negative ionization mode (LC2). Solvent A (2000 mL water + 4 mL formic acid) and solvent B (2000 mL acetonitrile + 4 mL formic acid) were used as eluents. Gradient LC1: 0–0.25 min (0.8 mL/min, 0% B), 0.25–1.5 min (0.8 mL/min; 0–12% B), 1.5–2.7 min (0.8 mL/min; 12–17.5% B), 2.7–4 min (0.8 mL/min; 17.5–50% B), 4–4.5 min (0.8 mL/min; 50–100% B), 4.5–4.7 min (0.8–1 mL/min; 100% B), 4.7–5 min (1 mL/min; 100% B), 5–5.1 min (1 mL/min; 100–0% B), 5.1–5.8 min (1–0.8 mL/min; 0% B). Gradient LC2: 0–0.25 min (0.8 mL/min, 0% B), 0.25–0.5 min (0.8 mL/min; 0–25% B), 0.5–2 min (0.8 mL/min; 25–50% B), 2–3 min (0.8 mL/min; 50–75% B), 3–3.5 min (0.8 mL/min; 75–100% B), 3.5–4.7 min (0.8–1 mL/min; 100% B), 4.7–5 min (1 mL/min; 100% B), 5–5.1 min (1 mL/min; 100–0% B), 5.1–5.8 min (1–0.8 mL/min; 0% B). For quantification and quality assurance, the MetIDQ software was used according to the manufacturer’s instructions.

### 2.6. Statistical Analysis

Percentage body weight changes of each animal relative to its day 0 (starting administration period) were used for statistical analysis and group comparison to fit a mathematically reasonable comparison avoiding varying initial body weight in the different groups, which were obtained by always dividing by the body weight on day 0 for each animal. Body weights, organ weights, and blood thyroid hormone levels between different groups were tested using the Mann-Whitney U test in Python (Scipy, version: 1.5.0).

The tissue metabolomics samples, i.e., thyroid gland and liver, were measured by targeted metabolomics with included internal standards as described in the previous paragraph. Thus, the expression of each measured metabolite was represented as absolute concentration (μM). Only the metabolites identified in at least 3 replicates out of 5 were considered reliable and selected for further analysis. Principal component analysis (PCA) analysis was carried out with the log2 transformed concentration values and tested with the PERMANOVA method in Python (Scikit-bio, version: 0.5.6). And the fold change (FC) on volcano plots were calculated based on absolute concentrations: mean(treatments)/mean(controls) and expressed as log2(FC). It should be noted that untreated animals with the same feeding cycle as the treated group were always selected as the control group. *p*-values were calculated with the Mann-Whitney U test and adjusted with Benjamini-Hochberg (BH) adjustment method using the included functions in R (version: 4.0.3). The results were included in the Supplementary File with sheet name Tissue.

When analyzing the plasma metabolomics data set, we adopted the above workflow with a few modifications. Briefly, since the absolute concentration values were normalized and expressed as fold changes, we, therefore, applied one more normalization step on the fold changes before the fold change and *p*-value calculation, which is called variance stabilization normalization in R (DEP, version 1.12.0). And metabolites identified in at least 7 replicates out of 10 were considered reliable and selected for further analysis. And the results were included in the Supplementary File with the sheet name Plasma.

To summarize our findings, heatmaps were generated for each drug under investigation at both the clinical index and metabolite class levels. We employed a similar strategy for calculating fold change (FC) values as described earlier with minor adjustments. For each clinical index, FC was computed using absolute values such as weights or concentrations: median(treatment)/median(control), which were then transformed logarithmically(log2). Notably, fold changes in body weights (BWs) were determined through the calculation of the difference between the treatment group’s median value and the control group’s median value without any logarithmic transformation due to their representation as relative body weight in this research. Additionally, the heatmaps displayed solely significant alterations with insignificant ones being depicted in gray color. For each metabolite class, we computed fold change by taking the average of all significantly altered metabolites’ FC values.

All plots were made with Python (version: 3.8.3) scripts that were written in-house, and Inkscape (version: 1.1).

## 3. Results

### 3.1. Body Weight (BW) Changes

**Figure 1:**
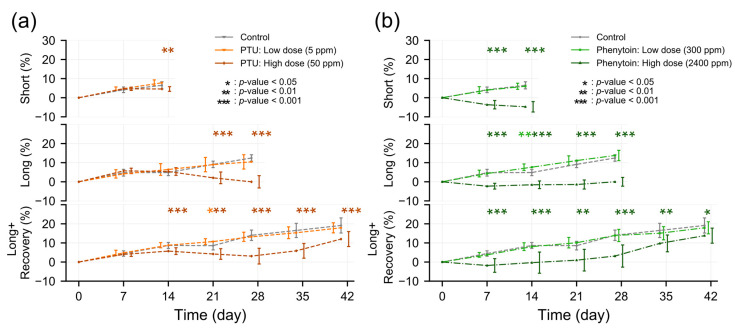
Weekly body weight changes. The percentage change in body weight in animals administered either PTU (**a**) or phenytoin (**b**) compared to non-treated control animals. Values are median body weight percentage change compared to baseline values from day 0 with standard deviations (SDs). Significance was calculated by the Mann-Whitney U test, with *: *p* < 0.05, **: *p* < 0.01, ***: *p* < 0.001.

To make the comparison between experiment groups more straightforward, we analyzed the change in BW to the baseline day 0 (Figure 1a,b). The untreated control animals had an increase in relative body weight for the experiment of 6.5% (range 3.3~10.2%) on day 13, and this reached 12.5% (range 9.2~15.2%) on day 27 and increased to 19.1% (range 12~26.9%) on day 41 (Figure 1a,b). The animals administered PTU or phenytoin at a low dose did not reveal any significant difference in the change in BW compared to the control animals for the experiment. However, for the high-dose PTU treatment, we observed significantly smaller values in relative body weight increase at day 13 (median 4.6%, range 1.9~5.9%, *p* = 0.007) and day 27 (median 0%, range −6.1~6.4%, *p* < 0.001). This persisted after the two-week recovery phase, where the increase in relative BW was lower for those animals which had received PTU at high doses compared to the untreated control animals (median 12%, range 4.8~15.7%, *p* < 0.001). A similar pattern was observed for the animals receiving the high-dose treatment of phenytoin. Here, we also observed a decrease in relative BW compared to the untreated controls after 13 days (median −4.8%, range −8.7~−0.3%, *p* < 0.001) and 27 days (median 0%, range −3.7~2.7%, *p* < 0.001), while as after the two-week recovery phase, the relative BW was increased less than for the control (median 13.8%, range 9.4~22.4%, *p* = 0.036).

### 3.2. Thyroid Hormone Blood Concentrations

**Figure 2:**
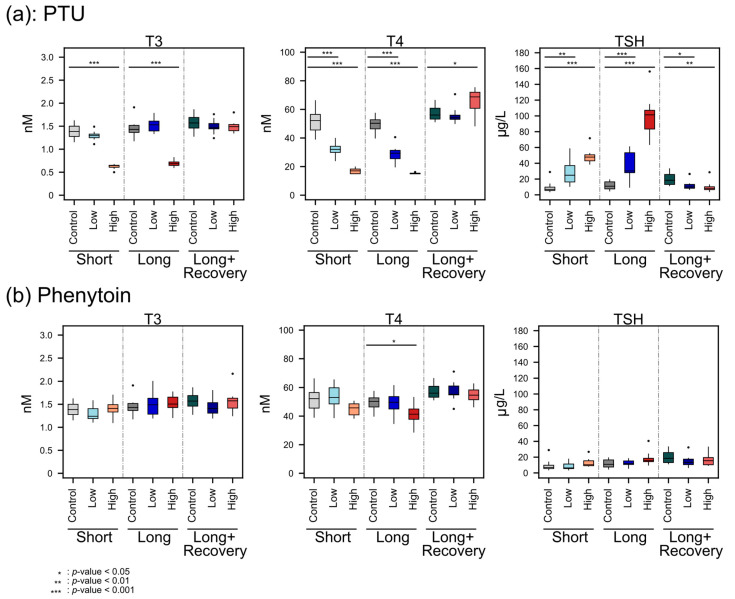
Plasma concentration of T3, T4, and TSH changes after PTU (**a**) and Phenytoin (**b**) exposure at high and low doses compared to controls for short-term and long-term exposure and after the recovery phase. Significance was calculated by the Mann-Whitney U test, with *: *p* < 0.05, **: *p* < 0.01, ***: *p* < 0.001.

For PTU treatment (Figure 2a), we observed a significant decrease in T3 levels after short exposure (PTU: median 0.65 nM, range 0.5~0.67 nM; control: median 1.39 nM, range 1.16~1.62 nM; *p* < 0.001) as well as after long exposure (PTU: median 0.69 nM, range 0.6~0.82 nM; control: median 1.43 nM, range 1.18~1.91 nM; *p* < 0.001) to the high dose of the drug, while no significant differences in plasma T3 concentration were observed for the low-dose drug exposure when compared to the control. These T3 levels recovered to normal after the recovery phase (PTU recovery: median 1.49 nM, range 1.35~1.8 nM; control: median 1.57 nM, range 1.28~1.86 nM; *p* = 0.353), i.e., post PTU administration. For the thyroid hormone T4, we observed a similar pattern with the high dose of PTU significantly decreasing T4 plasma levels after short exposure (PTU: median 17.01 nM, range 15~19.79 nM; control: median 52.2 nM, range 39.14~66.11 nM; *p* < 0.001) and long exposure (PTU: median 15 nM, range 15~16.18 nM; control: median 50.29 nM, range 39.89~57.37 nM; *p* < 0.001). Interestingly, we also observed, for the low dose of PTU, a significant decrease in plasma T4 levels after short- (PTU: median 32.04 nM, range 24.08~39.79 nM; *p* < 0.001) and long-term (PTU: median 29.37 nM, range 19.74~40.57 nM; *p* < 0.001) exposure. After the recovery phase, T4 levels for the low dose of PTU reverted to similar levels as the control (PTU: median 54.11 nM, range 50.05~70.62 nM; control: median 56.06 nM, range 51.3~66.35 nM; *p* = 0.481), while after the recovery phase for the high-dose treatment, the T4 levels were significantly higher than the control (PTU: median 68.74 nM, range 48.42~75.23 nM; *p* = 0.043). To get a better insight into this observation, we also measured TSH levels in plasma samples, the hormone regulating T3 and T4 synthesis. Both for the low- and the high dose of PTU, TSH levels were significantly increased after short- (low dose: median 24.85 μg/L, range 10.59~58.45 μg/L, *p* = 0.001; high dose: median 47.86 μg/L, range 38.75~71.72 μg/L, *p* < 0.001; control: median 7.02 μg/L, range 3.84~28.94 μg/L) as well as long-term exposure (low dose: median 30.3 μg/L, range 9.57~60.93 μg/L, *p* < 0.001; high dose: median 101.36 μg/L, range 63.64~156.29 μg/L, *p* < 0.001; control: median 10.92 μg/L, range 4.79~19.19 μg/L). A striking finding was that after the recovery phase, TSH levels did not revert to the same levels as the control but were lower than the control (median 18.49 μg/L, range 11.47~33.13 μg/L) after the low dose (median 9.93 μg/L, range 6.72~26.42 μg/L, *p* = 0.019) and high-dose (median 8.08 μg/L, range 4.03~28.63 μg/L, *p* = 0.002) administration of PTU. This might be explained by the sensitive negative feedback regulation, where the T4 level significantly increased.

Neither T3 nor TSH levels were affected by low- or high-dose administration of Phenytoin (Figure 2b). The same was generally true for T4, except for high-dose treatment after long-term administration, where a slight significant decrease in plasma T4 levels was observed (high dose: median 41.36 nM, range 28.76~53.13 nM; control: median 50.29 nM, range 39.89~57.37 nM; *p* = 0.019).

### 3.3. Morpho-Histological Changes in the Thyroid Gland

**Figure 3:**
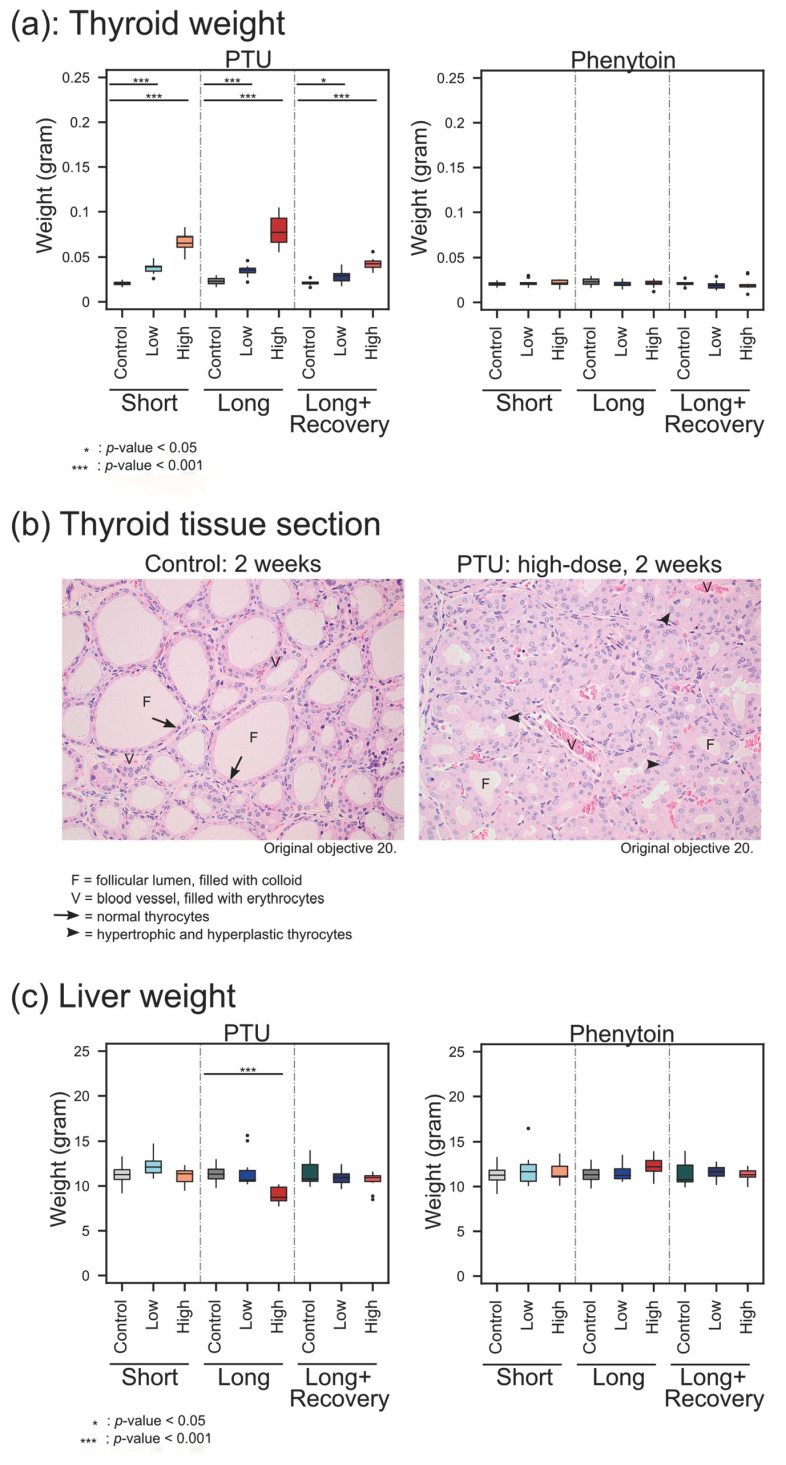
Thyroid weight (**a**), histological images of the thyroid (**b**), and Liver weight (**c**). Organ weights are shown for controls and after PTU and Phenytoin exposure at high or low doses with short-term and long-term exposure as well as an additional recovery phase. Significance values were calculated by the Mann-Whitney U test, with *: *p* < 0.05, ***: *p* < 0.001.

The thyroid gland weight increased after short-term exposure to the low dose of PTU compared to the control (low dose: median 0.039 g, range 0.026~0.048 g, control: median 0.021 g, range 0.017~0.024 g, *p* < 0.001) (Figure 3a). The increase was also observed after long-term exposure (low dose: median 0.0355 g, range 0.022~0.046 g; control: median 0.023 g, range 0.017~0.029 g, *p* < 0.001). For the high-dose PTU exposure, there was an even higher increase in thyroid gland weight compared to the control after short-term exposure (high dose: median 0.066 g, range 0.048~0.082 g; *p* < 0.001) as well as the long-term exposure (high dose: median 0.077 g, range 0.056~0.104 g, *p* < 0.001). Interestingly, After the recovery phase, there was still a significant increase in thyroid gland weight for the animals treated with the low dose (median 0.029 g, range 0.018~0.041 g, *p* = 0.0147) as well as the high dose (median 0.043 g, range 0.033~0.056 g, *p* < 0.001) compared to the control (median 0.022 g, range 0.016~0.027 g). 

Histopathological changes on thyroid follicular epithelial cells after administration were observed (Figure 3b). As can clearly be seen (Figure 3b), the H&E-stained section of a healthy rat thyroid gland shows thyroid gland follicles lined by a layer of low cuboidal thyrocytes. The follicular lumen was filled with clear colloids. However, upon PTU exposure, thyrocytes increased in size and number and showed a piling up of epithelial cells, whereby the follicular lumen became smaller. These findings represent severe diffuse follicular hypertrophy and hyperplasia of thyroid glands (Figure 3b).

Phenytoin treatment did not alter the thyroid gland weight (Figure 3a) nor showed any histopathologic changes when compared to thyroids from untreated animals.

### 3.4. Drug-Associated Changes in the Thyroid Metabolome

**Figure 4:**
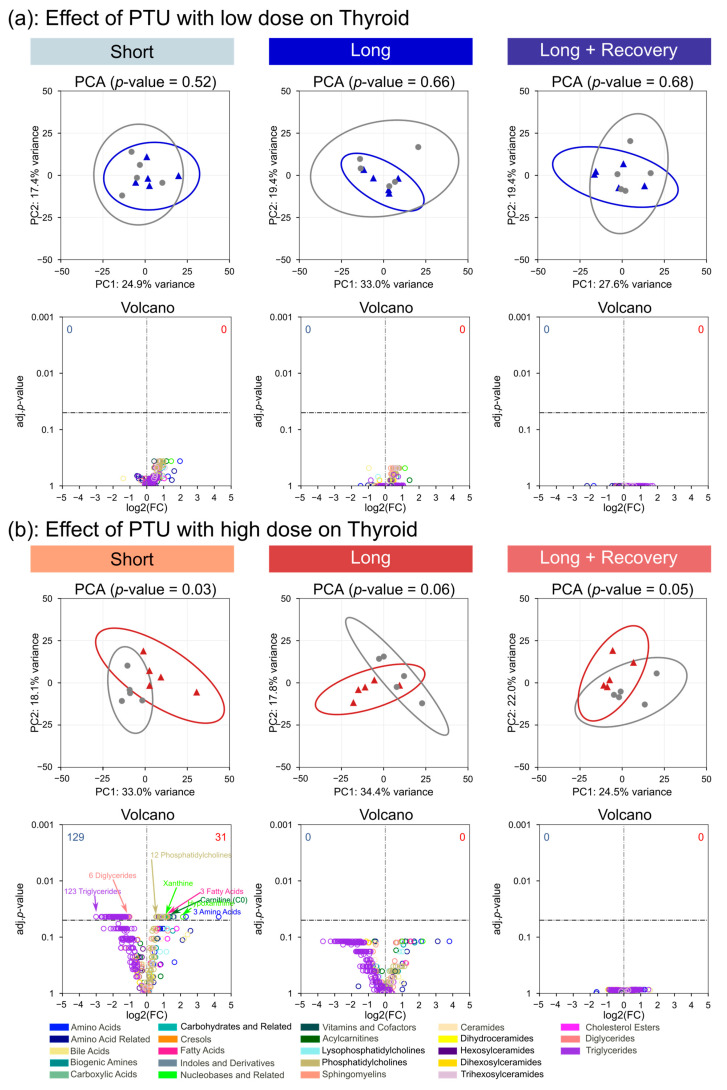
Low- (**a**) and high-dose (**b**) PTU-associated changes on the metabolome of the thyroid gland after short-term (2 weeks) and long-term drug administration (4 weeks), as well as an additional 2-week recovery phase after long-term exposure. Differences in global metabolome profiles between treatments (blue and red representing low and high doses, respectively) to controls (grey) were analyzed by PCA, with their significance determined by PERMANOVA. Differences in metabolite concentrations are visualized in a volcano plot depicting log2 fold changes of treatment versus control with their significance calculated by the Mann-Whitney U test and *p*-values corrected for multiple testing using the Benjamini-Hochberg method.

To investigate if drug-induced changes in circulating thyroid hormone levels were associated with alterations in the thyroid, a targeted analysis of metabolites from thyroid tissue was performed. In total, 490 metabolites from 23 metabolite classes were identified and quantified in the thyroid tissue (Supplemental Table S1). No significant global metabolic changes associated with short- or long-term, low-dose PTU treatment, as examined by principal component analysis, were observed (Figure 4a). Also, no significant changes in concentration for any single metabolite were seen during the low-dose PTU treatment. However, when PTU was administrated at a high dose, significant global changes in the global metabolite profile were seen after short exposure to PTU (*p* = 0.03), with a no longer significant separation observed after long-term exposure (*p* = 0.06) and after the recovery phase (*p* = 0.05). On the metabolite level, we observed 160 metabolites with a significant change in concentration after short-term exposure to the high dose of PTU compared to the control. A total of 129 of those metabolites were observed with a decrease in concentration. These were mainly triglycerides (123 compounds) and some diglycerides (6 compounds). Of the 31 metabolites observed at increased concentrations after short-term, high-dose exposure to PTU, the majority were still lipids. Specifically, of these, 12 were phosphatidylcholines, 3 fatty acids (arachidonic acid; eicosadienoic acid (20:2); eicosapentaenoic acid), and carnitine (log2 fc: 1.38; *p* = 0.043). Non-lipid metabolites observed at increased concentrations included three amino acids, namely cysteine (log2 fc: 4.26; *p* = 0.043), glutamate (log2 fc: 2.33; *p* = 0.043), and glycine (log2 fc: 0.88; *p* = 0.043). The two nucleobases-related metabolites, xanthine (log2 fc: 1.08; *p* = 0.043) and hypoxanthine (log2 fc: 2.14; *p* = 0.043) were also seen at increased concentrations.

Interestingly, after long-term exposure to the high dose of PTU, no metabolites with significantly altered concentration to the control were observed. As expected, after the recovery phase, no single metabolite was observed at significantly altered concentrations at low- or high-dose PTU exposure compared to the control (Figure 4a,b).

Phenytoin administration at low- or high-dose revealed no significant changes in the global metabolic profile of the thyroid tissue (Figure S1a,b), nor were any single metabolites observed with significant changes in concentration compared to the control.

### 3.5. Drug-Associated Morpho-Histological Changes and Metabolomic Changes in the Liver

Liver weights during low-dose PTU treatment were not significantly different in the liver from the untreated control animals over all time points (Figure 3c). Only after long-term exposure during a high-dose PTU administration was a significant decrease in liver weight observed when compared to the controls (high dose: median 8.725 g, range 7.79~10.11 g, control: median 11.315 g, range 9.85~12.93 g, *p* < 0.001).

**Figure 5:**
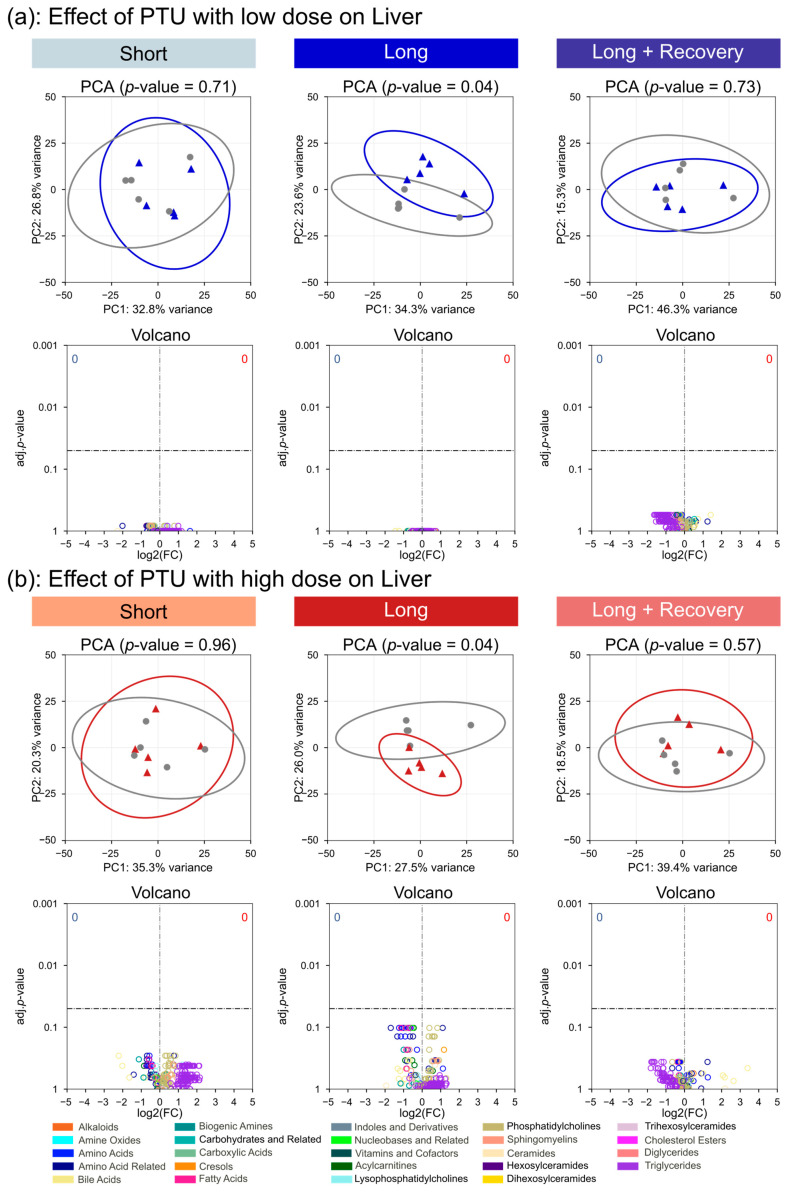
Low- (**a**) and high-dose (**b**) PTU-associated changes on the metabolome of the liver after short-term (2 weeks), long-term drug administration (4 weeks), and following a 2-week recovery phase after long-term exposure. Differences in global metabolome profiles comparing treatment (colored) to control (grey) analyzed by PCA with significance determined by PERMANOVA. Differences in metabolite concentrations visualized in volcano plot depicting log2 fold changes of treatment versus control with significance calculated by the Mann-Whitney U test and *p*-values corrected for multiple testing using the Benjamini-Hochberg method.

For the PTU treatment at a low dose, there was barely any influence on the metabolism of liver tissue. Global metabolite profile was not significantly altered after short-term treatment compared to the control (*p* = 0.71), though after long-term treatment, there was a slightly significant difference observed (*p* = 0.04) (Figure 5a). On the metabolite level at either short- or long-term, low- or high-dose PTU treatment, no single metabolite was observed with a significant change in concentration compared to the control. After the recovery phase for both low- and high-dose PTU, there was no significant difference in global metabolite profile compared to the control, nor was a single metabolite significantly altered in concentration.

Phenytoin administration, either at low or high doses, did not alter the global metabolic profile (Figure S2a,b). This was further seen on the metabolite level, where no single metabolite was detected at significantly different concentrations after phenytoin treatment compared to the control (Figure S2a,b).

### 3.6. Drug-Associated Changes in the Plasma Metabolome

**Figure 6:**
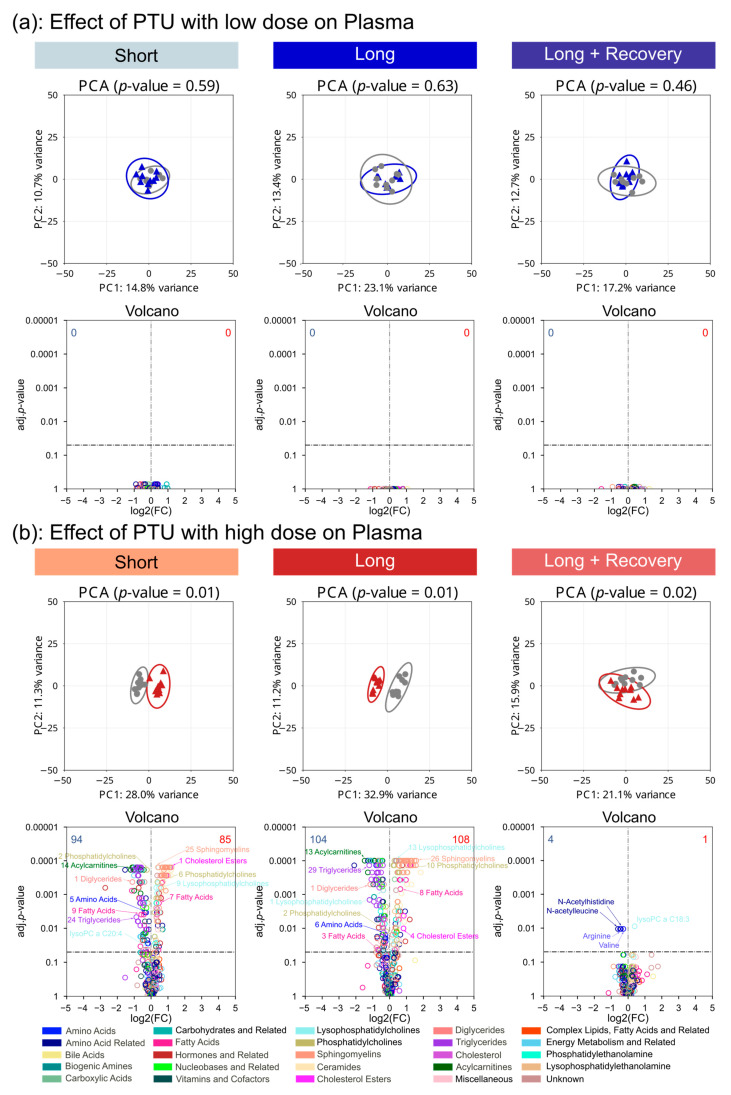
Low- (**a**) and high-dose (**b**) PTU- associated changes on the plasma metabolome after short-term (2 weeks), long-term drug administration (4 weeks), and following a 2-week recovery phase after long-term exposure. Differences in global metabolome profiles comparing treatment (colored) to control (grey) analyzed by PCA with significance determined by PERMANOVA. Differences in metabolite concentrations visualized in volcano plot depicting log2 fold changes of treatment versus control with significance calculated by the Mann-Whitney U test and *p*-values corrected for multiple testing using the Benjamini-Hochberg method.

To determine if the drugs induced systemic effects, we investigated the plasma metabolome. During low-dose PTU exposure, no significant difference after short-term or long-term exposure (Figure 6a) was observed for the plasma metabolic profiles. In addition, at low doses after PTU treatment, no single metabolite was seen at significantly altered concentrations (Figure 6a). However, high-dose PTU administration did result in a marked significant difference compared to the control in the short-term exposure group (*p* = 0.01), as well as in the long-term exposure group (*p* = 0.01) (Figure 6b). Even after the recovery phase, a significant difference in the metabolomes was seen (*p* = 0.02) (Figure 6b). On the metabolite level, this was already observed after short-term exposure to the high dose of PTU (Figure 6b). Accordingly, 179 and 212 metabolites were significantly altered in concentration in the short-term and long-term, respectively. In particular, in the short-term exposure group, 5 were down-regulated amino acids, i.e., alanine (log2 fc: −0.21; *p* = 0.028), glycine (log2 fc: −0.26; *p* = 0.011), threonine (log2 fc: −0.29; *p*= 0.003), isoleucine (log2 fc: −0.18; *p*= 0.028), and glutamate (log2 fc: −0.7; *p* < 0.001); 14 were down-regulated acylcarnitines; 24 were down-regulated triglycerides, and 1 was down-regulated diglycerides, i.e., DG(18:1_18:2) (log2 fc: −0.11; *p* < 0.001), among others. In the long-term exposure group, 6 down-regulated amino acids were found, i.e., glutamate (log2 fc: −0.79; *p* < 0.001), glycine (log2 fc: −0.29; *p* = 0.02), isoleucine (log2 fc: −0.45; *p* = 0.001), leucine (log2 fc: −0.25; *p* = 0.015), threonine (log2 fc: −0.4; *p* < 0.001), and valine (log2 fc: −0.24; *p* = 0.024). In addition, 13 acylcarnitines, 29 triglycerides, and one diglyceride were found to be downregulated. The metabolite identified as up-regulated after short-term, high-dose exposure to PTU included many lipids such as 25 sphingomyelins, 9 lysophosphatidylcholines, and 6 phosphatidylcholines. This up-regulation in lipids persisted in the long-term exposure to the high dose of PTU after long-term, high-dose exposure to PTU including many lipids such as 26 sphingomyelins, 13 lysophosphatidylcholines, and 10 phosphatidylcholines observed at significantly higher concentrations than for the control.

After the recovery phase, nearly all metabolites were observed as similar to the control, with only a few exceptions. These included arginine (log2 fc: −0.48; *p* = 0.011), valine (log2 fc: −0.28; *p* = 0.011), N-acetyl histidine (log2 fc: −0.43; *p* = 0.011) and N-acetyl leucine (log2 fc: −0.6; *p* = 0.011) were observed at decreased concentration compared to the control.

During low-dose short-term administration of phenytoin, we did not observe any significant difference in global metabolome profiles to the control groups in the short-term exposure group, as well as in the long-term exposure (Figure S3a). For the low-dose phenytoin exposure, no single metabolite was detected at different concentrations as the control (Figure S3a). Upon high-dose administration, we observed a significant separation of global metabolome profiles in the long-term exposure group (Figure S3b). On the single metabolite level, we observed 9 and 34 metabolites significantly changed, in the short-term and long-term, respectively. Specifically, in the short-term exposure group, one acylcarnitine and one sphingomyelin were at decreased concentrations, i.e., (hexadecanoylcarnitine (C16), log2 fc: −0.52; *p* = 0.042; SM(d32:2), log2 fc: −0.53; *p* = 0.042), and one amino acid was observed at increased concentration, i.e., histidine (log2 fc: 0.32; *p* = 0.04). In the long-term exposure group, we detected 7 acylcarnitines which were downregulated, while 2 triglycerides, 4 lysophosphatidylcholines, 4 phosphatidylcholines, and 2 fatty acids metabolites were found to be upregulated. Moreover, after the recovery phase, no metabolite was detected with a significantly different concentration from the control.

## 4. Discussion

Several studies have investigated the mechanism of hypothyroidism through different experimental designs. These included human plasma [29], as well as rat models [15,16,30]. A number of drugs like methimazole [16], PTU [15,16,30], and phenytoin [31] have been tested in adopted dosages [15] and exposure courses [15,32]. This study, in comparison, was designed to follow metabolomics alterations in a dose- and time-dependent rat model that was exposed to two different drugs, namely PTU, which directly influences TH synthesis in the thyroid [10], and phenytoin, which influences the excretion of THs from the body [13]. In this study, both PTU and phenytoin altered the TH levels and affected body weight. Although we observed large changes in the plasma metabolome induced by PTU and phenytoin, on the tissue level, we only observed changes in the metabolome in the thyroid during high-dose exposure to PTU. Nevertheless, the findings of this study should enhance the understanding of how the metabolome is affected by these drugs, and the additional two weeks of recovery should highlight changes in the metabolome are reversible.

The decreasing T4 concentrations with the higher PTU dose administration indicated the hypothyroid effect of PTU, which may have facilitated a change in energy metabolism and, therefore, a change in body weight. PTU is known to reduce TH levels by inhibiting thyroid TPO and thereby blocking thyroid hormone synthesis, as well as inhibiting the conversion of T4 to T3 by deiodinase type 1 (D1) in peripheral tissues, which is the predominate source of T3 [3,10]. However, T3 plasma levels were not decreased during low-dose administration of PTU, possibly because PTU concentrations may have been too low in peripheral tissue. A previous study also reported no effect of PTU on T3 blood levels in low concentrations (0.1 mg/kg/day vs 0.2 mg/kg/day in our study) [32]. However, when a high dose of PTU was administered, T3 decreased significantly, which is also in line with the findings of O’Connor et al. [32].

Furthermore, the TH feedback regulation was observed as expected since, with lower THs concentrations, significantly higher TSH concentrations were detected. Moreover, after the recovery phase, the TSH concentration was even lower than the controls. This might be explained by the sensitive negative feedback regulation, where the T4 level significantly increased. Furthermore, the smaller weight gain of high-dose PTU-administered animals compared to the untreated control animal was also in line with previous studies on rats [33,34].

The plasma metabolome of the rats only changed significantly after high-dose administration of PTU, suggesting that the low-dose administration of PTU had little effect on the thyroid. This was further seen for the metabolomes of the thyroid tissue and liver, where the low dose of PTU had no significant impact on metabolite concentrations. In contrast, the changes in metabolome for high-dose PTU administration were already considerable after short-term exposure and were further observed after long-term exposure. Interestingly we observed decreasing triglycerides and acylcarnitines concentrations in the plasma. Decreases in triglyceride blood concentrations have been previously observed in rats [21,34,35,36], which contrasts with observations in humans [36]. Triglycerides are important energy sources, and in blood, triglycerides enable the bidirectional transfer of adipose fat and glucose from the liver [37]. It is widely known that THs regulate fatty acid transportation and metabolism. Lower blood triglyceride levels are probably caused by lower activity of lipoprotein lipases [38]. Adipose lipoprotein lipases are increased in rats deficient in thyroid hormone [39]. In humans, T4 levels were associated with acylcarnitine concentrations in plasma, suggesting that T4 could influence *β*-oxidation and cellular fatty acid transport [40].

Furthermore, in the study, an increase in phosphatidylcholines was observed. Our findings revealed plasma lower T4 levels with a decrease in acylcarnitines and an increase in phosphatidylcholines, including lysophosphatidylcholines and sphingomyelins, matching the previously published results. Interestingly, in the thyroid tissue at short-term exposure to the high dose of PTU, the decrease in many triglycerides and an increase in many phosphatidylcholines also suggested the same mechanistic hypothesis. In addition, we observed at short-term high PTU administration higher concentration of carnitine, which could hint at lower levels of carnitine being loaded with acyl moieties, resulting in a decrease in *β*-oxidation activity. Though, there seemed to be an adaptation of the thyroid tissue to the lower levels of THs, since, after long-term exposure to high-dose PTU, no lipids in the organ tissue were significantly altered in concentration compared to the control animals. A further interesting finding in the thyroid was the increase in the concentration of the two fatty acids, arachidonic acid and eicosapentaenoic acid, with known metabolic signaling functions. Contrary to most thyroid metabolomes, both these fatty acids were seen at higher concentrations at short-term, high-dose PTU exposure. Especially, eicosapentaenoic acid has been found to decrease cardiometabolic disease risk and levels of circulating triglycerides [41]. This would suggest that the higher eicosapentaenoic acid in the thyroid might directly contribute to the lower TGs concentrations in plasma and thyroid after high-dose PTU treatment.

Amino acids also exhibited significant changes in concentration when exposed to high-dose PTU. Interestingly, all the amino acids with a significant change were downregulated. This was observed in the plasma already after short-term administration of PTU and was further seen after long-term exposure in the plasma and the liver. One study described a decrease in glutamate during hypothyroidism, which we observed as well, and an increase in tyrosine, tryptophan as well as arginine [21]. In our study, arginine, tryptophan, and tyrosine were not significantly changed in concentration in the plasma during short and long-term administration of PTU. THs are known to control lipid metabolism, and TH levels are lowered during hypothyroidism, possibly indicating that amino acids are increasingly used as an energy source to compensate for the decrease in lipid utilization through *β*-oxidation. Hence, this could lead to the observed decreasing amino acid concentrations. Another potential reason is that amino acids are not being released to the same level from endogenous proteins, which was previously reported in a study investigating rat skeletal muscle [42], where a lack of THs led to reduced protein breakdown. A study with human participants [43], however, did not find any associations between T4 hormone levels and amino acid concentrations in plasma. However, T3 levels were not analyzed. Potential support for this hypothesis was provided by a study where hyperthyroidism was induced through the application of T3 in rats [44]. Therein, Short et al. showed that elevated T3 levels lead to an increased turnover of proteins, including both their synthesis and breakdown. In our study, only in the PTU-treated animals did we observe a decrease in amino acids. Phenytoin administration did not alter T3 levels in the plasma. Taken together, this implies that in our study, the decrease in amino acids was probably associated with a decrease in T3 and not T4.

Phenytoin administration only led to significant changes in plasma T4 levels at high doses, though the effect size was a lot smaller than that for PTU. Neither the plasma concentrations for T3 nor for TSH were significantly altered, which may explain the rather scarce effect on the metabolomes of the thyroid, plasma, and liver, respectively. A previous study found significantly decreased levels of T4 and increased levels of TSH in human serum samples from patients treated long-term with phenytoin [45]. The T4 findings fit our results. However, we did not observe an increase in TSH. This could be because the other study involved human patients.

Interestingly, after the recovery phase, the metabolomes of the thyroid, liver, and plasma were generally indistinguishable from controls for both drugs tested at high and low doses, suggesting that the observed effects were reversible.

The current study provides valuable results to clarify the effects of drug-induced hypothyroidism on molecular perturbations on the metabolome in plasma, thyroid, and liver tissue in a rat model system. The data were collected using a rat model system. Even though the rat is a proven and accepted model organism, there are species-specific differences that do not allow for a one-to-one transferability of the results to humans, as already mentioned above. The study did not measure the internal exposure levels of the two drugs, though estimates were made based on the calculated drug intake by rats. The tissue metabolome was measured using the MxP Quant 500 kit. However, there is no previous evidence to support the use of this kit on tissue samples, despite the manufacturer’s claim that it is suitable for serum, plasma, urine, and feces. The presented study focuses solely on a metabolomics-based view and given the surprisingly marginal effects that have been observed in most treatment groups, we assume that an additional transcriptomics or proteomics layer would enhance the study, i.e., to evaluate any adaptation in gene expression or protein abundance to counter any metabolic changes from the drugs. The investigation of metabolic fluxes might also be highly beneficial in order to analyze how these drugs affect metabolite turnover rates. This could be performed using stable isotopically labeled substrates to follow fluxes, though this would be expensive to set up in a rat model.

## 5. Conclusions

**Figure 7:**
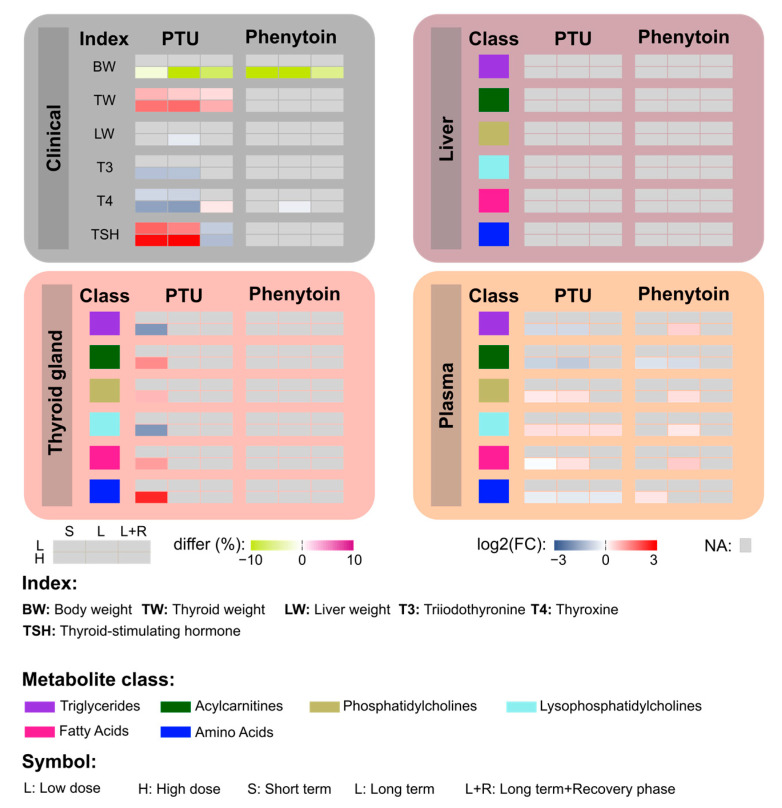
Summary of clinical outcomes and the most significantly changed metabolites treated with PTU and Phenytoin. In this section, four boxes are presented, outlining the main findings, including six measured clinical indexes and six focused classes of metabolites. For each index/class, the 2 heatmap boxes on the right summarize the regulatory directions in each treatment group. Particularly, only significant changes were presented here, while non-significant changes were marked as NA (grey color).

In conclusion, both drugs decreased T4 plasma concentrations. However, only high-dose PTU administration significantly lowered T3 and activated TSH plasma concentrations (Figure 7). For PTU, we observed more clinical changes than for Phenytoin but no metabolic effects in liver samples. Thus, we could not observe direct effects on the liver. In contrast, we observed metabolic changes in the thyroid after exposure to the higher concentration of PTU. And we also found metabolic changes in plasma samples both for PTU and Phenytoin. Thus, this study indicates that the effects of PTU can be detected by metabolomics.

## Data Availability

The plasma metabolomics measurement result data set was already uploaded into Zenodo with a DOI link: https://doi.org/10.5281/zenodo.5900664. The tissue metabolomics datasets are available at the NIH Common Fund’s National Metabolomics Data Repository (NMDR) website, the Metabolomics Workbench [46], https://www.metabolomicsworkbench.org where it has been assigned Study ID ST002023. The data can be accessed directly via its Project DOI: http://dx.doi.org/10.21228/M8MD8N.

## Supplementary Materials

The following supporting information can be downloaded at: https://www.mdpi.com/article/10.3390/metabo13070847/s1, Figure S1: Low (a) and high-dose (b) phenytoin-associated changes on the metabolome of the thyroid gland after short-term (2 weeks) and long-term drug administration (4 weeks), as well as an additional 2-week recovery phase after long-term exposure; Figure S2: Low (a) and high-dose (b) phenytoin-associated changes on the metabolome of the liver after short-term (2 weeks) and long-term drug administration (4 weeks), as well as an additional 2-week recovery phase after long-term exposure; Figure S3: Low (a) and high-dose (b) phenytoin-associated changes on the plasma metabolome after short-term (2 weeks) and long-term drug administration (4 weeks), as well as an additional 2-week recovery phase after long-term exposure; Table S1: Metabolites were detected and quantified in different sample types.
