# Supplementary material for: Assessing the Influence of Propylthiouracil and Phenytoin on the Metabolomes of the Thyroid, Liver, and Plasma in Rats
Source: Metabolites. 2023 Jul 14;13(7):847. doi: 10.3390/metabo13070847 (PMC10383188; doi:10.3390/metabo13070847)
Supplement: Supplementary file 1 [file metabolites-13-00847-s001.zip › metabolites-2296454_Supplementary Materials.pdf]

Supplementary Materials:

Table S1. Metabolites were detected and quantified in different sample types.

Metabolites classes	Thyroid gland	Liver	Plasma
Alkaloids		1	
Amine Oxides		1	
Amino Acids	20	19	20
Amino Acid Related	18	23	44
Bile Acids	8	11	2
Biogenic Amines	6	7	4
Carbohydrates and Related	1	1	19
Carboxylic Acids	1	5	1
Cresols	1	1	
Fatty Acids	7	5	34
Hormones and Related			26
Indoles and Derivatives	2	4	
Nucleobases and Related	2	2	12
Vitamins and Cofactors	1	1	15
Acylcarnitines	14	12	14
Lysophosphatidylcholines	9	10	22
Phosphatidylcholines	65	48	18
Sphingomyelins	15	9	27
Ceramides	26	12	13
Dihydroceramides	2		
Hexosylceramides	16	3	
Dihexosylceramides	8	1	
Trihexosylceramides	6	1	
Cholesterol Esters	4	2	6
Diglycerides	24	8	1
Triglycerides	234	145	32
Cholesterol			1
Complex Lipids, Fatty Acids and Related			9
Energy Metabolism and Related			16
Miscellaneous			12
Lysophosphatidylethanolamine			7
Phosphatidylethanolamine			16
Unknown			37
Total	490	332	408

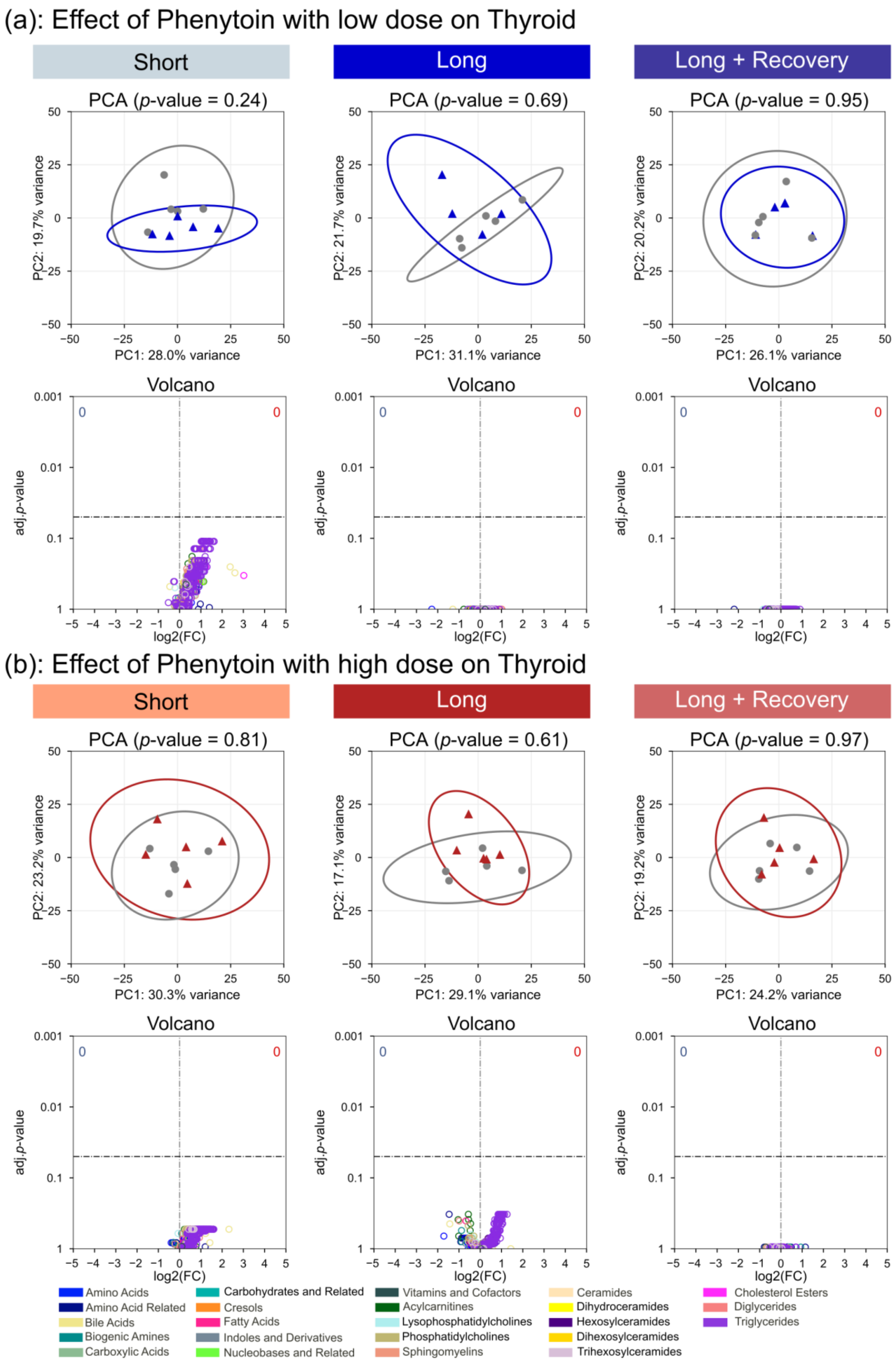

(a): Effect of Phenytoin with low dose on Thyroid
Short
Long
Long + Recovery
PCA (p-value = 0.24)
PCA (p-value = 0.69)
PCA (p-value = 0.95)
PC2: 19.7% variance
PC1: 28.0% variance
PC2: 21.7% variance
PC1: 31.1% variance
PC2: 20.2% variance
PC1: 26.1% variance
Volcano
adj.p-value
log2(FC)
(b): Effect of Phenytoin with high dose on Thyroid
Short
Long
Long + Recovery
PCA (p-value = 0.81)
PCA (p-value = 0.61)
PCA (p-value = 0.97)
PC2: 23.2% variance
PC1: 30.3% variance
PC2: 17.1% variance
PC1: 29.1% variance
PC2: 19.2% variance
PC1: 24.2% variance
Volcano
adj.p-value
log2(FC)
Amino Acids
Amino Acid Related
Bile Acids
Biogenic Amines
Carboxylic Acids
Carbohydrates and Related
Cresols
Fatty Acids
Indoles and Derivatives
Nucleobases and Related
Vitamins and Cofactors
Acylcarnitines
Lysophosphatidylcholines
Phosphatidylcholines
Sphingomyelins
Ceramides
Dihydroceramides
Hexosylceramides
Dihexosylceramides
Trihexosylceramides
Cholesterol Esters
Diglycerides
Triglycerides

Figure S1. Low (a) and high-dose (b) phenytoin-associated changes on the metabolome of the thyroid gland after short-term (2 weeks) and long-term drug administration (4 weeks), as well as an additional 2-week recovery phase after long-term exposure. Differences in global metabolome profiles between treatments (blue and red representing low and high-dose, respectively) to controls (grey) were analyzed by PCA, with their significance determined by PERMANOVA. Differences in metabolite concentrations are visualized in a volcano plot depicting log2 fold changes of treatment versus control with their significance calculated by the Mann-Whitney U test and P-values corrected for multiple testing using the Benjamini-Hochberg method.

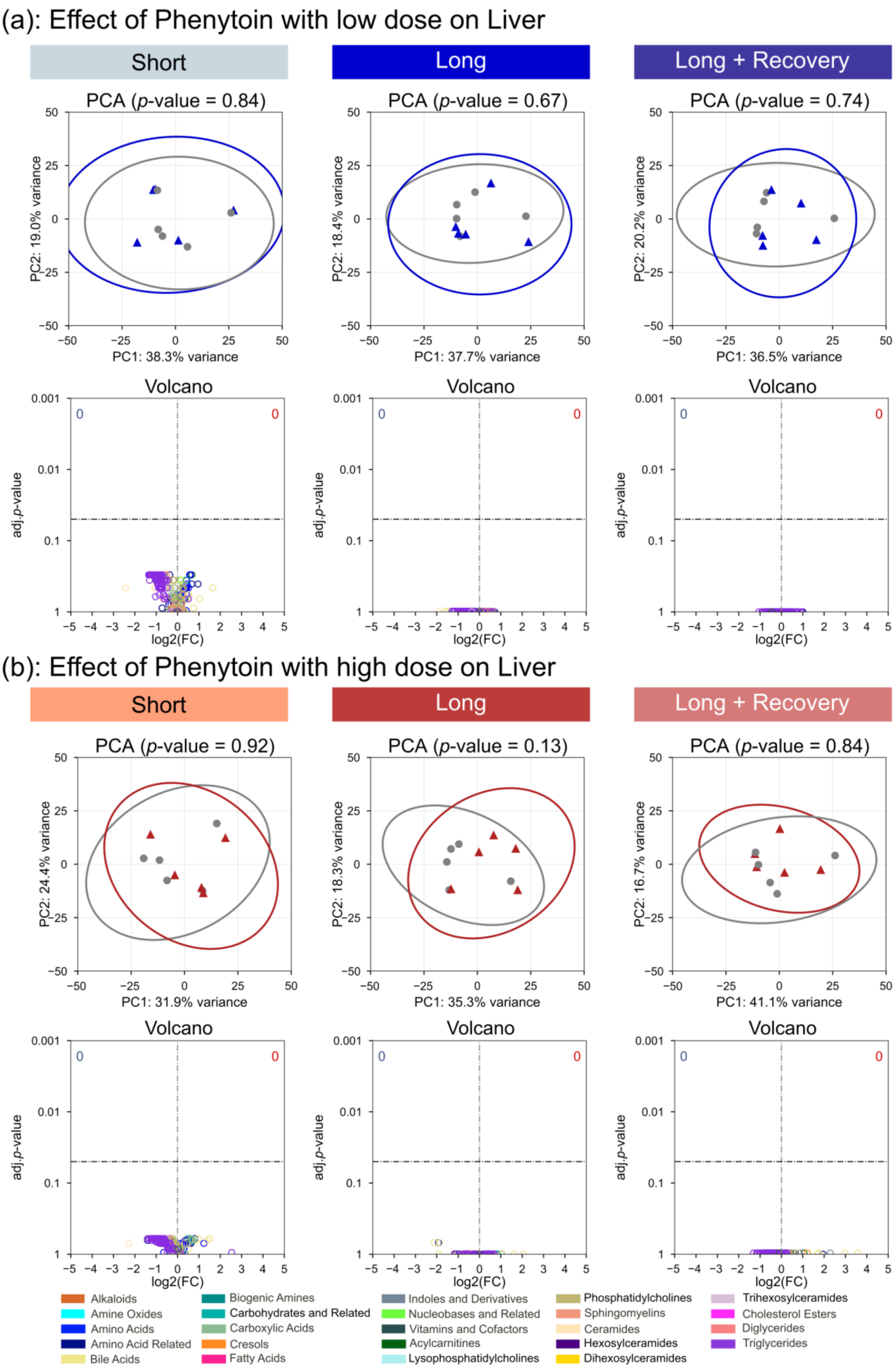

Figure S2. Low (a) and high-dose (b) phenytoin-associated changes on the metabolome of the liver after short-term (2 weeks) and long-term drug administration (4 weeks), as well as an additional 2-week recovery phase after long-term exposure. Differences in global metabolome profiles between

treatments (blue and red representing low and high-dose, respectively) to controls (grey) were analyzed by PCA, with their significance determined by PER-MANOVA. Differences in metabolite concentrations are visualized in a volcano plot depicting log2 fold changes of treatment versus control with their significance calculated by the Mann-Whitney U test and P-values corrected for multiple testing using the Benjamini-Hochberg method.

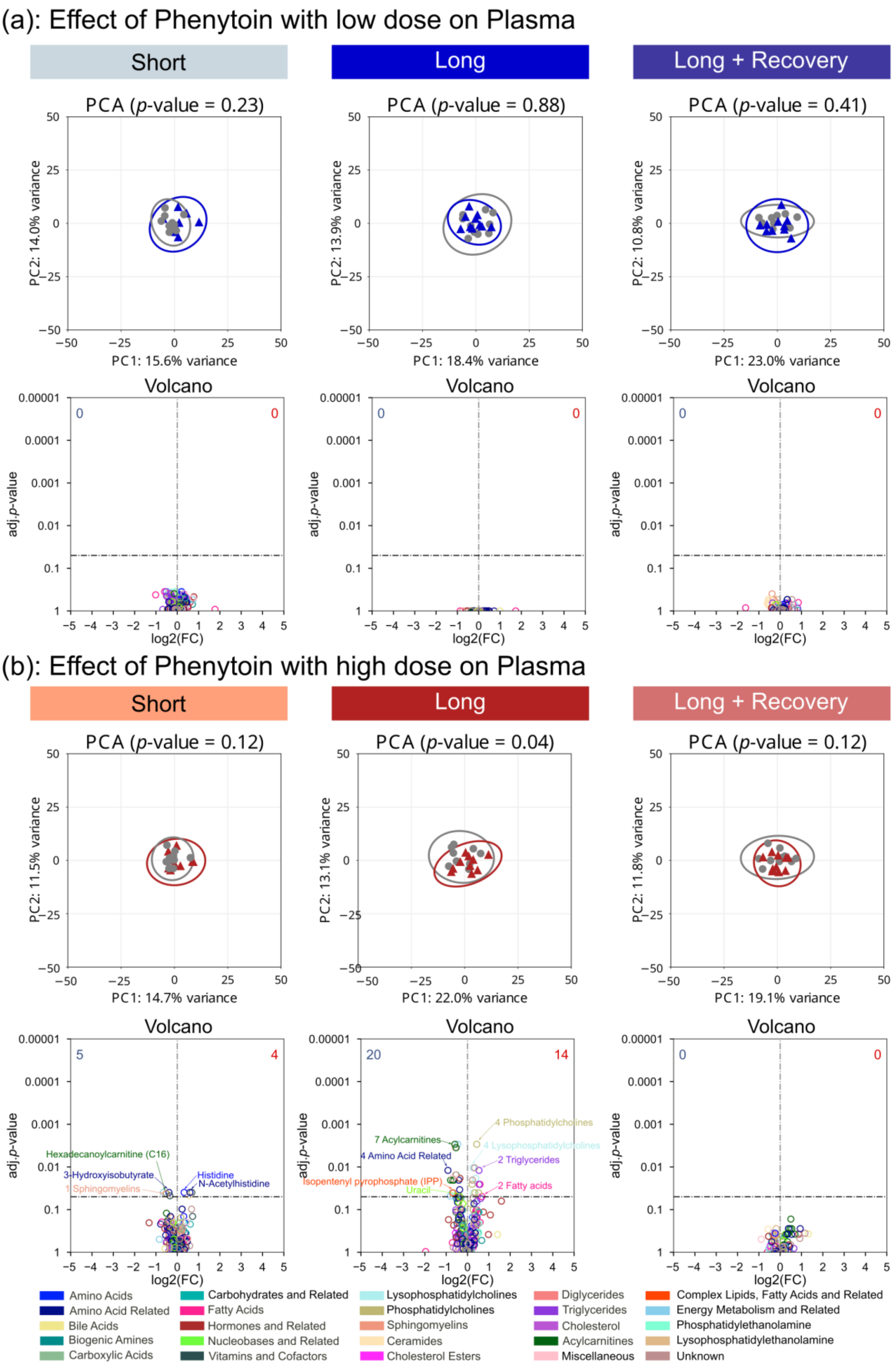

Figure S3. Low (a) and high-dose (b) phenytoin-associated changes on the plasma metabolome after short-term (2 weeks) and long-term drug administration (4 weeks), as well as an additional 2-week recovery phase after long-term exposure. Differences in global metabolome profiles between

treatments (blue and red representing low and high-dose, respectively) to controls (grey) were analyzed by PCA, with their significance determined by PERMANOVA. Differences in metabolite concentrations are visualized in a volcano plot depicting log2 fold changes of treatment versus control with their significance calculated by the Mann-Whitney U test and P-values corrected for multiple testing using the Benjamini-Hochberg method.
